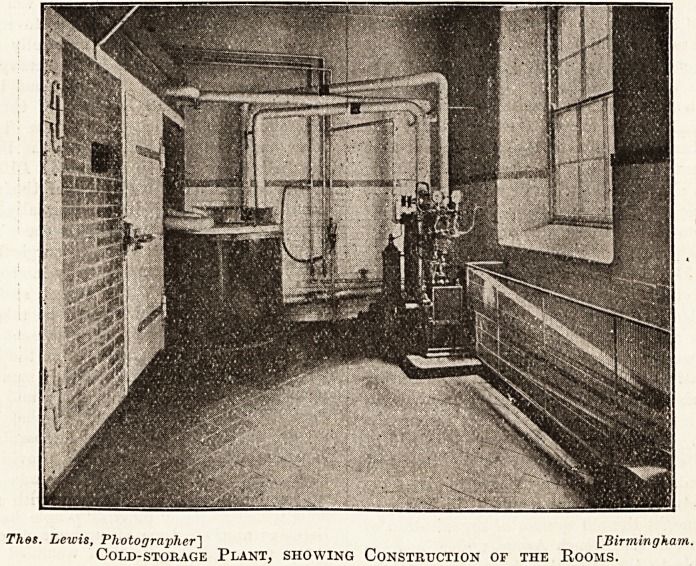# Home-Made Hospital Equipment: A Cold-Storage Plant at a Provincial Hospital

**Published:** 1915-03-20

**Authors:** 


### HOME-MADE HOSPITAL EQUIPMENT.

### A Cold-Storage Plant at a Provincial Hospital.

Only few hospitals have a cold-storage plant, and the approach of summer always brings inquiries. We give below a detailed description, a plan, and photograph, together with the cost, of a plant for a hospital of 300 beds. The work of construction and erection was carried out by the hospital's own works department.

Plan of Cold-storage Plant.

A disused store room, 20 feet by 20 feet, was utilised for the purpose of the installation, half the space being adapted for the cold rooms and half for the machinery. There are two cold rooms, one for milk and butter (kept at a temperature of 42 degrees), and another room for meat and poultry (kept at a temperature of 38 degrees).

### The Machinery Employed.

The refrigerating machinery is of the Linde type, $CO_2$ being the medium employed. It consists of motor, compressor, refrigerator, pump, two brine tanks. The motor is of the direct current type of Westinghouse make, 3 horse-power, complete with starter, etc. The current is obtained from the Corporation Tramways.

The compressor, which contains the $CO_2$ gas and is driven by the motor, is rather a complicated piece of mechanism. It is fitted with a valve for taking a supply of gas from an ordinary cylinder, an internal chamber with suction and delivery valves that operate when compressing the gas, and an outer chamber through which water passes for cooling the compressed gas.

The refrigerator is a large vessel having an outer shell of teak and an inner tank of galvanised iron, with a cavity of 2 inches filled with granulated cork.

The pump is of the rotary type, driven from the same motor.

The brine tanks are of galvanised iron.

To lower the temperature of the rooms and make the ice the motor is started, and the gas comes under pressure in the compressor. It escapes down the delivery pipe to the coil in the refrigerator, the brine in which is lowered at once. In the centre of the coil of pipes in the refrigerator are two galvanised boxes, which contain the blocks of ice which are thus manufactured.

The pump is also started and the brine begins to circulate from the tanks in each room over the

*Thos. Lewis, Photographer*] [*Birmingham.*
Cold-storage Plant, showing Construction of the Rooms.

$CO_2$ coil in the refrigerator, thus causing the temperature of each room to drop at once.

The Construction of the Rooms.

The rooms are 8 feet in height. The top is covered by a concrete slab, the front is of 4½-inch pressed brickwork in cement, the whole of the rooms being lined with 4 inches of cork (two 2-inch slabs), the joints being crossed in every case. The slabs of cork are fastened to the walls by hot bitumen, and are fastened to each other by the same material.

The division wall is of 4-inch cork, with a light steel framework to support it in the centre of the two slabs.

The floor is of 4-inch cork (two 2-inch slabs), and finished with ¾ inch thick rock asphalt, the asphalt being also continued 6 inches up the walls.

The walls are finished in Parian cement put directly on to the cork; the whole is then painted with three coats of paint and one coat of enamel, which gives a smooth and clean appearance.

During the work of construction the importance of having no needless projections and no dust or dirt traps, whilst having all tank covers and air-circulating ducts movable and easy to clean, was kept constantly in view.

The cost of the machinery, construction, and internal fittings was £270.

## Figures and Tables

**Figure f1:**
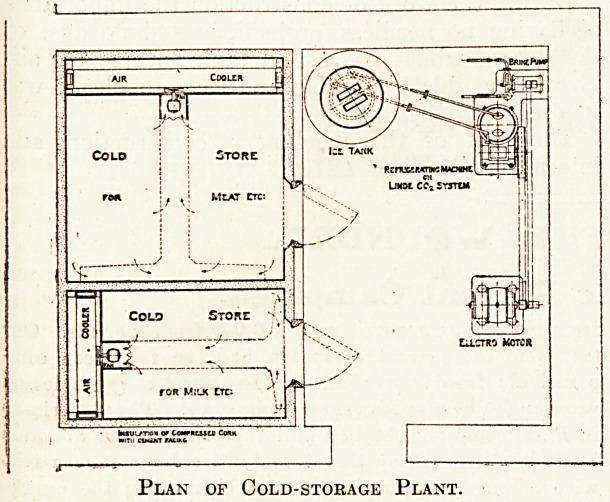


**Figure f2:**